# Influence of intergenerational *in utero* parental energy and nutrient restriction on offspring growth in rural Gambia

**DOI:** 10.1096/fj.201700017R

**Published:** 2017-08-04

**Authors:** Kamilla G. Eriksen, Elizabeth J. Radford, Matt J. Silver, Anthony J. C. Fulford, Rita Wegmüller, Andrew M. Prentice

**Affiliations:** * Medical Research Council (MRC) Elsie Widdowson Laboratory, Cambridge, United Kingdom;; † Cambridge University Hospitals National Health Service (NHS) Foundation Trust, University of Cambridge, Cambridge, United Kingdom; ‡ Department of Genetics, University of Cambridge, Cambridge, United Kingdom;; § Medical Research Council (MRC) Unit, The Gambia, Banjul, Gambia;; ¶ Medical Research Council (MRC) International Nutrition Group, London School of Hygiene and Tropical Medicine, London, United Kingdom

**Keywords:** epigenetics, birth season, parental influences, fetal and postnatal growth

## Abstract

The prenatal environment can alter an individual’s developmental trajectory with long-lasting effects on health. Animal models demonstrate that the impact of the early life environment extends to subsequent generations, but there is a paucity of data from human populations on intergenerational transmission of environmentally induced phenotypes. Here we investigated the association of parental exposure to energy and nutrient restriction *in utero* on their children’s growth in rural Gambia. In a Gambian cohort with infants born between 1972 and 2011, we used multiple regression to test whether parental season of birth predicted offspring birth weight (*n* = 2097) or length (*n* = 1172), height-for-age *z* score (HAZ), weight-for-height *z* score (WHZ), and weight-for-age *z* score (WAZ) at 2 yr of age (*n* = 923). We found that maternal exposure to seasonal energy restriction *in utero* was associated with reduced offspring birth length (crude:−4.2 mm, *P* = 0.005; adjusted: −4.0 mm, *P* = 0.02). In contrast, paternal birth season predicted offspring HAZ at 24 mo (crude: −0.21, *P* = 0.005; adjusted: −0.22, *P* = 0.004) but had no discernible impact at birth. Our results indicate that periods of nutritional restriction in a parent’s fetal life can have intergenerational consequences in human populations. Fetal growth appears to be under matriline influence, and postnatal growth appears to be under patriline intergenerational influences.—Eriksen, K. G., Radford, E. J., Silver, M. J., Fulford, A. J. C., Wegmüller, R., Prentice, A. M. Influence of intergenerational *in utero* parental energy and nutrient restriction on offspring growth in rural Gambia.

Early life represents a critical window of phenotypic plasticity, and nutritional stressors during this period alter growth and development, resulting in permanent changes in metabolism and chronic disease susceptibility (1, 2). Substantial seasonal nutritional fluctuations are experienced naturally in some rural farming communities in low- and middle-income countries, resulting in maternal undernutrition in the hungry season (3, 4). In Gambia, a chronically marginal diet in rural areas is aggravated by a hungry season, when food stocks from the previous harvest season are depleted and the burden of laborious seasonal farm work and exposure to infectious diseases is higher. In women, this results in an average seasonal weight loss of 3–6 kg, and the weight gain of pregnant women during the hungry season is 400–500 g/mo less than the weight gain during the harvest season (5, 6). Infants born in the hungry season are exposed to these nutritional stressors in mid-to-late fetal life, resulting in a lower birth weight compared with infants born in the harvest season and a higher mortality from infectious diseases in young adulthood (7–9). Similar associations have been reported in other rural farming communities (4, 10, 11). Our previous work has demonstrated that maternal nutrition around conception is associated with lasting changes in the epigenetic profile of certain genes, which may contribute to the season-of-birth disparity in health outcomes (12, 13).

There is strong evidence from animal models that the impact of nutrition in early life can extend over multiple generations and that the early-life environment of both parents is important for the health of offspring (2, 14–17). There are at least two potential mechanisms for such nonmendelian phenotypic inheritance: recapitulation of an altered environment (*e.g.*, through altered maternal gestational metabolism) and epigenetic inheritance (18, 19). The latter is strongly implicated where paternal transmission of environmentally induced phenotypes is observed (14, 16, 17, 20–22). The developing germline, which was previously thought to prevent intergenerational epigenetic inheritance in mammals, undergoes extensive epigenetic remodeling *in utero*. However, recent studies have demonstrated that a substantial number of regions in the human germline escape epigenetic erasure and thus may be candidates for intergenerational epigenetic inheritance (23–26). It is unclear whether specific regions systematically and reliably escape erasure in humans.

Opportunities to explore the intergenerational effects of gestational energy and nutrient restriction are rare in human populations, and consequently data are sparse. There is evidence that the offspring of women exposed *in utero* to famine have a greater risk of poor health in later life than the offspring of unexposed women (27) and that paternal famine exposure is associated with increased body mass index in adulthood (28). A study investigating the impact of famine in China in the early 1960s suggest that children whose parents were born during the famine were shorter compared with children with neither parent born during famine. No association was found for exposure of a single parent (29). Furthermore, a historical study of 3 generations in an isolated community in northern Sweden has reported sex-specific associations between abundant harvests during grandparental prepubertal growth and the mortality risk of grand-offspring (30–32). However, these Swedish data have been criticized for having small sample sizes and being subject to bias and confounding (33). There is a need for further high-quality epidemiologic studies in this area. In this study in rural Gambia, we investigated the association between paternal and maternal early nutritional conditions according to seasonality and their offspring’s fetal and postnatal growth.

## MATERIALS AND METHODS

Residents from 3 villages (Keneba, Kantong Kunda, and Manduar) of the West Kiang District in Gambia were included in this retrospective study. The data for this study were derived from birth records and recorded family data going back 43 yr. Birth weights and birth lengths were recorded within 72 h of birth by trained staff, regardless of whether the mother gave birth at the Medical Research Council facility or in her village. Birth weights from the 3 villages were recorded to the nearest 10 g with a Salter spring balance and tarred sling, which was calibrated regularly. Trained staff conducted regular anthropometric measurements of the offspring, from which the values at 24 ± 3 mo have been selected for this analysis.

Environmental conditions in Gambia are characterized by a long, hot, and dry season from November until May and a shorter wet season from June until October (5). Due to the harvest season from September to December, food supply is plentiful from November to May. The harvest does not cover food needs for the entire year, and a hungry season develops in June and lasts until October (5). There is low food quantity, as well as food quality, during this season, with a reduced intake of several nutrients (34). The effects of food shortages are exacerbated by the high workload required to generate the next year’s crops. This study investigates the exposure to energy and nutrient restriction in late gestation by defining parents born between July and December as having been exposed to intrauterine undernutrition in mid- to late gestation (henceforth referred to as the hungry season) and between January and June as unexposed (henceforth referred to as the harvest season). These cutoffs for seasonality have previously been used in studies of intrauterine effects in this Gambian population (6, 8, 9). To separately examine influences of offspring fetal and postnatal growth, outcome measures considered were birth weight and length, height-for-age *z* score (HAZ), weight-for-age *z* score (WAZ), and weight-for-height *z* score (WHZ) at 2 yr of age. *Z* scores were calculated using the World Health Organization Anthro program (v.3.2) against the World Health Organization 2006 growth standard.

The potential confounding variables investigated were maternal age, parity, parental height, gestational age at birth, offspring sex, offspring season of birth, and season of offspring anthropometric measurements at 24 mo of age. Interactions of potential confounding variables, including offspring sex, were also investigated, but no interactions were found. Offspring month of birth and month of measurement were included in the multiple regressions using Fourier terms (35). The use of Fourier terms allows the decomposition of any periodic function into a linear combination of simple oscillating functions (sines and cosines) parameterized by coefficients (the Fourier coefficients) (35). In this analysis, 6 sets of Fourier terms were used (3 pairs of sines and cosines).

Simple stratification, using *z* test and linear regression for hypothesis testing, was performed to examine the crude association between outcome variables (offspring growth) and exposure variables (parental season of birth). Any crude associations between the outcome and exposure variables, identified with a value of *P* ≤0.05, were taken forward for multiple regression.

Potential confounding variables were added to the multiple regressions one at a time. All statistical analyses were performed with STATA version 14.

## RESULTS

We identified 812 unique mothers and 447 unique fathers for whom season of birth was known. On average, fathers had 4 children (range, 1–22) and mothers had 3 children (range, 1–13), resulting in 2829 mother–offspring pairs and 1609 father–offspring pairs. There were considerable missing data for some variables, as shown in **Table 1**, resulting in reduced sample sizes for the final outcome measurements (**Table 2**). In the multivariate analyses of offspring birth weight and maternal birth season, the excluded subjects had a slightly lower mean birth weight than the included subjects (*P* = 0.05). In the multivariate analyses of birth length and maternal birth season, the excluded subjects did not have a different mean birth length compared with the included subjects. In the analysis of HAZ and paternal birth season, there was no difference in HAZ at 24 mo of age between included and excluded subjects.

**TABLE 1.** Characteristics of the study population

Population characteristics	Sample size	Maternal season of birth		Total	Sample size	Paternal season of birth		Total
		January–June	July–December			January–June	July–December	
Offspring sex [n (%)]								
Female	1376	687 (47.0)	689 (50.4)	1376 (48.6)	795	422 (51.4)	373 (47.2)	795 (49.4)
Male	1453	776 (53.0)	677 (49.6)	1453 (51.4)	814	399 (48.6)	415 (52.7)	814 (50.6)
Offspring season of birth [n (%)]								
Harvest season: January–June	1463	716 (52.0)	747 (51.5)	1463 (51.7)	821	395 (51.2)	426 (50.8)	821 (51.0)
Hungry season: July–December	1366	661 (48.0)	705 (48.5)	1366 (48.3)	788	376 (48.8)	412 (49.2)	788 (49.0)
Offspring gestational age at birth (wk)	1184	38.96 ± 1.62	38.83 ± 1.54	38.90 ± 1.58	508	39.01 ± 1.57	38.74 ± 1.60^*a*^	38.87 ± 1.59
Offspring birth weight (kg)	2097	2.984 ± 0.40	2.949 ± 0.42^*a*^	2.967 ± 0.42	1155	2.973 ± 0.42	2.957 ± 0.42	2.957 ± 0.42
Offspring birth length (mm)	1172	496.6 ± 0.99	492.4 ± 1.12^*a*^	494.7 ± 0.75	756	493.8 ± 25.90	494.2 ± 26.23	494.0 ± 26.04
Offspring WAZ at 24 mo	1841	−1.497 ± 0.96	−1.499 ± 0.99	−1.498 ± 0.97	923	−1.400 ± 0.95	−1.460 ± 0.95	−1.430 ± 0.95
Offspring HAZ at 24 mo	1839	−1.675 ± 1.15	−1.691 ± 1.09	−1.682 ± 1.12	923	−1.494 ± 1.17	−1.703 ± 1.07^*a*^	−1.598 ± 1.12
Offspring WHZ at 24 mo	1839	−0.867 ± 1.00	−0.867 ± 1.02	−0.867 ± 1.01	923	−0.877 ± 0.97	−0.802 ± 1.01	−0.840 ± 0.99
Maternal age (yr)	2827	26.62 ± 6.87	26.20 ± 6.81	26.42 ± 6.85	1335	25.24 ± 5.90	24.83 ± 6.36	25.04 ± 6.13
Maternal height (mm)	2536	1597.1 ± 55.2	1595.3 ± 62.0	1596.2 ± 58.6	1342	1604.7 ± 62.1	1603.6 ± 54.2	1604.2 ± 58.5
Parity [n (%)]								
Primiparous	812	408 (27.9)	404 (29.6)	812 (28.7)	555	278 (33.9)	277 (35.2)	555 (34.5)
Multiparous	2017	1055 (72.1)	962 (70.4)	2017 (71.3)	1054	543 (66.1)	511 (64.8)	1054 (65.5)
Paternal height (mm)	2119	1684.3 ± 53.3	1700.3 ± 57.8^*a*^	1692.2 ± 56.6	1124	1702.8 ± 66.1	1694.1 ± 57.0^*a*^	1698.3 ± 61.6

Data are are presented as means ± sd unless otherwise noted. *^a^*Evidence for a difference between parental seasons of birth, *P* ≤ 0.05.

**TABLE 2.** Crude and adjusted regressions of parental season of birth and offspring birth weight, birth length, and HAZ

Exposure/outcome	Association	Sample size	Coefficient	95% CI	*P*
Maternal season of birth/Offspring birth weight (kg)	Crude association between maternal season of birth and offspring birth weight	2097	−0.036 ± 0.018	−0.071 to −0.001	0.05
	Adjusted for offspring sex	2097	−0.029 ± 0.018	−0.064 to −0.005	0.09
	Adjusted for offspring season of birth*^a^*	2097	−0.036 ± 0.018	−0.071 to −0.001	0.04
	Adjusted for parity	2097	−0.034 ± 0.018	−0.069 to −0.0001	0.05
	Adjusted for maternal height	1991	−0.295 ± 0.018	−0.065 to −0.006	0.1
	Adjusted for paternal height	1683	−0.037 ± 0.020	−0.075 to −0.002	0.07
	Adjusted for all of the above	1631	−0.025 ± 0.019	−0.063 to −0.012	0.2
Maternal season of birth/Offspring birth length (mm)	Crude association between maternal season of birth and offspring birth length	1172	−4.228 ± 1.494	−7.160 to −1.297	0.005
	Adjusted for offspring sex	1172	−4.027 ± 1.483	−6.936 to −1.118	0.007
	Adjusted for offspring season of birth*^a^*	1172	−4.156 ± 1.497	−7.092 to −1.220	0.006
	Adjusted for parity	1172	−3.907 ± 1.491	−6.832 to −0.983	0.009
	Adjusted for maternal height	1107	−4.274 ± 0.014	−7.296 to −1.252	0.006
	Adjusted for paternal height	920	−4.496 ± 1.722	−7.875 to −1.116	0.009
	Adjusted for all of the above	889	−4.033 ± 1.736	−7.441 to −0.626	0.02
Paternal season of birth/Offspring HAZ at 24 mo	Crude association between paternal season of birth and offspring HAZ at 24 mo	923	−0.209 ± 0.074	−0.353 to −0.064	0.005
	Adjusted for offspring sex	923	−0.197 ± 0.074	−0.342 to −0.053	0.007
	Adjusted for offspring season of measurement*^a^*	922	−0.208 ± 0.074	−0.352 to −0.063	0.005
	Adjusted for maternal height	860	−0.264 ± 0.070	−0.401 to −0.127	<0.001
	Adjusted for paternal height	702	−0.199 ± 0.084	−0.363 to −0.035	0.02
	Adjusted for all of the above	673	−0.220 ± 0.077	−0.371 to −0.069	0.004

Coefficient values are presented as means ± se. *^a^*Offspring season of birth and season of measurement were included in the multiple regression as Fourier terms

The fathers included in this cohort were born between 1946 and 1989, mothers between 1947 and 1995, and offspring between 1972 and 2011. Twenty-nine percent of the mothers were primiparous, with a mean ± sd age of 26 ± 6.9 yr (Table 1). Of a total of 2097 offspring, the mean ± sd birth weight was 2.967 ± 0.42 kg, with 10% being born with a low birth weight (<2500 g). The offspring mean ± sd WAZ (*n* = 1841), HAZ (*n* = 1839), and WHZ (*n* = 1839) values at 24 mo of age were –1.50 ± 0.97, –1.68 ± 1.21), and –0.87 ± 1.01, respectively. The prevalence of stunting (HAZ < 2) was 39%, indicative of chronic malnutrition over the study period of 43 yr. The proportions of wasting (WHZ < 2) and underweight (WAZ < 2) were 12 and 28% respectively.

### Birth weight

Birth weight was associated with maternal season of birth, with a difference of 36 g between the two seasons [95% confidence interval (CI), −0.071 to −0.001; *P* = 0.05] (Table 2). When adjusting for offspring sex, season of birth, parity, and parental height, the difference decreased to 25 g and was no longer significant (95% CI, −0.063 to 0.012; *P* = 0.2) (Table 2). Because maternal height could be considered to be on the causal pathway, we also considered a model adjusted for all the above potential confounders excluding maternal height. However, this did not materially alter the results (data not shown). We analyzed offspring birth weight against paternal season of birth and found no association between paternal birth season and offspring birth weight (Table 1).

An additional sensitivity analysis with the exposure variable (parental season of birth) expressed instead as a series of Fourier terms to reflect monthly variation was also conducted. This analysis produced results that were very similar for all outcome measurements to those obtained using the dichotomized (harvest *vs*. hungry season) variable (data not shown).

### Birth length

Despite the lower sample size and the greater difficulties of accurately assessing birth length in mothers’ homes, there was strong evidence that offspring birth length was shorter in hungry-season mothers, with a crude difference of −4.2 mm (95% CI, −7.160 to −1.297; *P* = 0.005) compared with harvest-season mothers (Table 2). When adjusting for offspring sex, season of birth, parity, and parental height, the difference was −4.0 mm (95% CI, −7.441 to −0.626; *P* = 0.02). There was no detectable association between length at birth and paternal birth season (Table 1).

### *Z* scores at 24 mo of age

In contrast to birth measures, there was no association between maternal season of birth and HAZ at 2 yr of age (Table 1), but paternal birth in the hungry season was strongly associated with a reduced HAZ in offspring at 2 yr of age, with a crude difference of −0.21 according to paternal season of birth (95% CI, −0.353 to −0.064; *P* = 0.005) (Table 2). Strong evidence for an association remained after adjusting for offspring sex, season of measurement, and parental height (adjusted coefficient, −0.220; 95% CI, −0.371 to −0.069; *P* = 0.004) (Table 2). Omitting the adjustment for paternal height did not materially alter the results (data not shown). The association was not adjusted for offspring season of birth because this variable was not associated with offspring HAZ at 24 mo of age. There was no evidence for an association between either parent’s season of birth and offspring WAZ or WHZ at 24 mo (Table 1).

None of the associations in Table 2 varied significantly by sex of the offspring (birth weight by sex interaction, *P* = 0.1; birth length by sex interaction, *P* = 0.7; and HAZ by sex interaction, *P* = 0.9).

## DISCUSSION

We present evidence that mothers who were exposed to energy and nutrient restriction in the latter part of their own fetal development (those born in the rainy/hungry season) give birth to babies that are slightly shorter than the babies of unexposed mothers. This impact is limited to fetal growth because there was no evidence of an association between maternal birth season and offspring postnatal growth measurements (WAZ, HAZ, and WHZ) at 24 mo of age. Although the observed crude difference in birth length was small (4.2 mm), the suggestion of a potential link between offspring birth length and maternal birth season is informative from a mechanistic point of view. It is known that birth size is strongly influenced by placental function (36), maternal physiology, and maternal size *via* uterine constraint (37). Adjustment for all available confounders (including parental height) attenuated the effect very slightly (to 4.0 mm), but it remained significant. In an unadjusted analysis there was also a modest effect on birth weight (−36 g), but this disappeared after adjustment for offspring season of birth, sex, parity, and parental height.

Our data are consistent with those of Lumey *et al.* (38), who found some evidence in women who lived through the Dutch Hunger Winter that famine exposures in the third trimester of gestation reduced the birth weight of first-born offspring. However, subsequent analyses of this population have not found an association of maternal undernutrition in any stage of pregnancy with the next generation’s birth weight (27, 38, 39).

The timing of paternal birth in relation to nutritional stressors predicted offspring HAZ at 24 mo of age with or without controlling for possible confounders (including parental height). No evidence for an association between paternal season of birth and offspring birth size, WAZ, and WHZ at 24 mo of age was found. Our work is consistent with a previous study in this Gambian population that demonstrated no impact of paternal birth season on the next generation’s neonatal anthropometry (40).

It is possible that an underlying association between paternal fetal environment and offspring fetal growth exists but is obscured by more dominant maternal placental and uterine influences on fetal growth and by the relatively smaller sample size available in this analysis. Indeed, several studies have found stronger maternal–offspring than paternal–offspring birth weight correlations (41, 42). Kuzawa and Eisenberg (41) found that maternal birth weight was a stronger predictor of offspring birth weight than paternal birth weight, suggesting a dominant maternal influence on fetal growth. However, *prima facie*, our data suggest that when the infant is no longer subject to maternal *in utero* constraints, paternal influences on growth potential are more easily discerned, and hence paternal prenatal nutrition is associated with postnatal but not fetal growth.

Epigenetic inheritance is a potential mechanism through which environmentally induced phenotypes may be inherited. It has been implicated particularly where the paternal transmission of environmentally induced phenotypes is observed (14, 16, 17, 20–22), as in animal models in which there are fewer confounding factors compared with the maternal germline. The developing germline undergoes extensive epigenetic remodeling *in utero* involving the erasure and, in male fetuses, the reapplication of epigenetic marks (23–26). This process may be sensitive to the nutritional environment. Indeed, rodent studies have demonstrated that undernutrition, even limited to late pregnancy, permanently alters the germline DNA methylome (16). Previous research in this Gambian population has shown the effects of season of conception on the DNA methylome in somatic tissue. However, the impact of *in utero* exposures on the germline epigenome is unknown (12, 13). It is theoretically possible that paternal undernourishment *in utero* may alter fathers’ behavior or social status, with consequences for his offspring’s growth, but this has not been empirically tested. Further work is needed to identify the mechanism by which paternal late-gestational nutritional restriction leads to impaired postnatal growth in offspring.

Although the effect sizes noted here based on parental season of birth are not large, they may be part of a wider constellation of prior-generation exposures that have important implications for interventions attempting to overcome growth and other developmental deficits in children in low-income settings. They might imply that children’s ability to benefit from additional nutrients, both *in utero* and postnatally, will be constrained by epigenetically inherited limitors. An alternate, more optimistic, and in our judgment more likely interpretation that is supported by the secular changes in growth observed in countries emerging from poverty, is that populations can and will benefit from interventions but do so over a longer timescale than a single generation. It is important to counsel patients in public health policies for 2 reasons: *1*) a failure to recognize intergenerational limitors can lead to unrealistic expectations of interventions (*e.g.*, in the first 1000 d) and therefore to a loss of confidence and investment, and *2*) attempts to override such limitations with higher and higher nutrient inputs can lead to metabolic disease in later life by imposing nutrient conditions that are not harmoniously tuned to the epigenetically entrained expectation of the nutrient (and other environmental) conditions to which an infant will be exposed throughout life.

An important strength of this study was the availability of high-quality data in an environment where seasonal nutritional restriction is experienced naturally in a repeating annual pattern. This provides a unique opportunity to explore the consequences of nutritional restriction *in utero* in a large population with comprehensive antenatal and child growth data collected over several decades. The data were of high quality with few inaccuracies, and field workers had received standardized training in data collection, increasing the validity and reliability of this study. This study had large sample sizes (minimum *n* > 650 for multivariable regression), which is unusual for studies of this type.

Limitations of this study include confounders that were not explored because the data were not available, including maternal birth weight and socioeconomic status, which are known to be associated with fetal growth (20, 40, 43). Missing data decreased the sample sizes in the multiple regressions, making them more prone to type 2 errors. However, the smallest sample size still exceeded 650 subjects. We found evidence for differences between included and excluded subjects in the analysis of offspring birth weight and maternal birth season: the excluded subjects had a lower mean birth weight than the included subjects. Furthermore, the measurements of offspring anthropometry at 24 mo of age were dependent on both routine visits and self-referral and are therefore vulnerable to selection bias. No technique was used to prove biologic paternity, which could have weakened the observed paternal associations. Our study assumes that the parents’ conceptions occurred randomly throughout the year and hence were not associated with a genetic variant that might affect both propensity to conceive at times of varying energy status and the size of offspring. We have searched for evidence of such an effect and have found none. Finally, there are several non-nutritional environmental factors that fluctuate seasonally, such as exposure to infection (8). Although we have characterized in detail the seasonal nutritional changes and have seen a strong correlation with outcomes (34), we cannot exclude some contribution from these other seasonal factors.

## CONCLUSIONS

Our data suggest that periods of nutritional restriction during both parents’ fetal life can have intergenerational consequences, affecting their offspring’s fetal and postnatal growth. We find that maternal birth in the hungry season is associated with reduced offspring birth length, whereas paternal birth in the hungry season is associated with reduced offspring HAZ at 24 mo of age. Further work is required to investigate the mechanisms through which the parental early life environment alters the developmental trajectory of their children. It is possible that early nutrition may influence maternal development and physiology, altering the maternal intrauterine environment, with consequences for offspring development. It is unclear whether parental nutritional restriction may alter behavioral or socioeconomic factors with consequences for offspring growth. Another intriguing possibility is that the epigenome of the developing parental germline, in flux in mid to late pregnancy, is altered by gestational nutritional status and contributes to altered offspring development. The developing germline has been shown to be vulnerable to nutritional compromise in lower mammals, but this hypothesis remains to be tested directly in a human population.

## Abbreviations

CI: confidence interval
HAZ: height-for-age *z* score
WAZ: weight-for-age *z* score
WHZ: weight-for-height *z* score
